# Supplementary material for: Chromothripsis during telomere crisis is independent of NHEJ, and consistent with a replicative origin
Source: Genome Res. 2019 May;29(5):737–49. doi: 10.1101/gr.240705.118 (PMC6499312; doi:10.1101/gr.240705.118)
Supplement: Supplemental Material [file supp_gr.240705.118_Supplemental_file_1.zip › contigs/annotated_contigs/DB53/contig.2.DB53_length_276_mean_cov_4.08333333333.docx]

**DB53_length_276_mean_cov_4.08333333333**

```
CATAAAAAACTTCCATCAAAAGGAATATGTATCTTGCTTCTTGGAAAGGGAAGTTTACTTTTCTGGGGCAGGGCTTAA|TGA|AGCTTT
 >chr8:125797613-125797691 - E=1e-35                                              >chr8
AAAAGAATTAGAAGCTGCTAAAACTGATTGGATTGTAGCTGTTTCCCTGAGACTTTGGTCTGGAATCTCAGGATATAAAGGTGCTAAGA
:135482680-135482875 + E=2e-106
GGATGAGGCCACATCCCAAAGCCCTTTCCACACAGTAGAAGTTGAGGCCCAGAGCAAAGAGGGGTGCCTCTAGGGTCACATGCAGAACC

AGGGGCAGAAT
```
